# Throughput assurance of wireless body area networks coexistence based on stochastic geometry

**DOI:** 10.1371/journal.pone.0171123

**Published:** 2017-01-31

**Authors:** Ruixia Liu, Yinglong Wang, Minglei Shu, Shangbin Wu

**Affiliations:** 1 College of Computer Science and Engineering, Shandong University of Science and Technology, Qingdao 2666590, China; 2 Shandong Computer Science Center (National Supercomputer Center in Jinan), Shandong Provincial Key Laboratory of Computer Networks, Jinan 250014, China; 3 Electronic and Computer Engineering School of Engineering and Physical Sciences, Heriot-Watt University, Edinburgh EH144AS, United Kingdom; West Virginia University, UNITED STATES

## Abstract

Wireless body area networks (WBANs) are expected to influence the traditional medical model by assisting caretakers with health telemonitoring. Within WBANs, the transmit power of the nodes should be as small as possible owing to their limited energy capacity but should be sufficiently large to guarantee the quality of the signal at the receiving nodes. When multiple WBANs coexist in a small area, the communication reliability and overall throughput can be seriously affected due to resource competition and interference. We show that the total network throughput largely depends on the WBANs distribution density (*λ*_*p*_), transmit power of their nodes (*P*_*t*_), and their carrier-sensing threshold (*γ*). Using stochastic geometry, a joint carrier-sensing threshold and power control strategy is proposed to meet the demand of coexisting WBANs based on the IEEE 802.15.4 standard. Given different network distributions and carrier-sensing thresholds, the proposed strategy derives a minimum transmit power according to varying surrounding environment. We obtain expressions for transmission success probability and throughput adopting this strategy. Using numerical examples, we show that joint carrier-sensing thresholds and transmit power strategy can effectively improve the overall system throughput and reduce interference. Additionally, this paper studies the effects of a guard zone on the throughput using a Matern hard-core point process (HCPP) type II model. Theoretical analysis and simulation results show that the HCPP model can increase the success probability and throughput of networks.

## Introduction

Population aging is poised to become a major issue in developing countries [1]. Wireless body area networks (WBANs) have broad application prospect for the healthy and aging population by providing health telemonitoring to caretakers [2]. Physiological signals such as body temperature, blood pressure, and electrocardiograms from an individual person can be monitored using a number of lightweight miniature sensors. Many applications that use WBANs for these purposes must maintain high reliability, energy efficiency, or spatial reuse ratio to meet strict requirements owing to the monitoring of disease signals [3].

WBANs play an important role in promoting the development of motion detection and mobile health. Related research mainly focuses on a single network, and very few studies deal with multiple WBANs. Because body movement is quite different from other types of motion, the dynamic topology changes of WBANs are also different from other wireless networks. Co-located WBANs occur when many people with wireless body sensor nodes coexist in a small area. In many cases, co-located WBANs result in interference or very strong competition among them for spectrum allocation [4]. Interference and resource competition significantly decrease network reliability, energy waste, and generally result in a decrease in the collective network throughput. When WBANs monitor critical health information, data loss can threaten patient lives [5]. Resource competition is one of the performance-limiting factors in coexisting wireless networks, leading to the decrease in system throughput. Consequently, maximizing the throughput of coexisting WBANs is critically important.

The field of stochastic geometry has developed a family of Poisson point process (PPP) distributions, which has been used in a variety of wireless network models over the last decade. PPP distributions provide a natural method of defining and computing critical network performance metrics such as interference distribution and outage probability by referring to specific random geometrical patterns for nodes [6].

The standards of wireless communication considered in WBANs are the IEEE 802.15.4 [7], IEEE 802.15.6 [8], and Bluetooth Low Energy [3]. IEEE 802.15.4 PHYs provides the capability to perform carrier-sensing multiple access with collision avoidance (CSMA-CA). Each node monitors the channel before transmission and defers the transmission if it senses a busy channel to avoid collision. To enhance the performance of CSMA-CA for IEEE 802.15.4, the authors of [9] proposed a collision-aware backoff algorithm. Some medium-access control (MAC) protocols create exclusion zones to protect scheduled transmissions. For example, Aloha creates a random exclusion disk around each node, which means that for an arbitrary radius around a node, a non-null probability exists that no nodes within this radius can transmit in a given time slot. At the same time, a sufficiently large random exclusion zone exists around all nodes [10].

Transmit power and carrier-sensing threshold are key parameters in CSMA wireless networks. Energy consumption is a challenging issue in WBANs because of the limited battery capacity in the sensor nodes. Therefore, effective power control is especially crucial in the design and performance of WBANs. The transmit power of sensor nodes should be related according to their changing surroundings to enhance energy efficiency and reduce conflicts with other devices, especially when many WBANs coexist. The carrier-sensing threshold is also a critical parameter that should be very carefully chosen to maintain a balance between frequency reuse efficiency and outage probability. The lower the carrier-sensing threshold is, the more the interference is reduced. However, a lower carrier-sensing threshold generally decreases the throughput. In this paper, we propose a joint carrier-sensing threshold and power control mechanism to meet the demands of coexisting WBANs based on the IEEE 802.15.4 standard using stochastic geometry. The minimum transmit power is derived according to the distribution of the coexisting networks and their carrier-sensing thresholds. The throughput is further improved by treating the transmitters of coexisting networks as uniformly distributed and optimizing their guard zone using a type II Matern hard-core point process (HCPP) model. In this study, the guard zone is defined as the hard-core size of the HCPP model. Theoretical analysis and simulation results show that these two methods can ensure total network throughput. In particular, this strategy decreases the total power consumption and enhances spatial frequency utilization.

Our research contributions are summarized as follows:

1. To reduce resource competition and collision in coexisting WBANs, this paper presents a new joint carrier-sensing threshold and power control mechanism according to the network distribution. We characterize the transmit power optimization, which depends on carrier-sensing threshold, and the density of coexisting WBANs.
2. Our second contribution is to derive closed-form expressions for the success probability and throughput. We compare the performance of coexisting IEEE 802.15.4-based WBANs using the proposed strategy within an HCPP model. Their difference and relationship are also analyzed.
3. We naturally decrease the interference and maximize the success probability by employing a guard zone around each node. This paper discusses the tradeoff between the throughput and guard zone size of coexisting WBANs using the HCPP model.

The remainder of this paper is organized as follows. The second part introduces the related work. The third part proposes and describes the system model. In fourth part, the joint carrier-sensing threshold and power control mechanism are proposed. The success probability and throughput are analyzed in detail where coexisting WBAN nodes are distributed according to a PPP model. The fifth part illustrates and analyzes the throughput of coexisting networks under an HCPP model. The sixth part compares our proposed strategy employing the PPP and HCPP models. The seventh part presents the evaluation of the performance of the proposed joint carrier-sensing threshold and power control strategy. Finally, the eighth part summarizes our work.

## Related Work

Coexisting WBANs is a typical application scenario because of the mobility and sociability of people. In the following discussions, we divide the related works into three sections. The first section is about network coexistence. The second section reviews the related works on power control mechanisms. The third section reviews the related work on throughput optimization.

### Network Coexistence

In the literature, most studies on coexisting networks, such as [11] and [12], are based on heterogeneous networks. However, the current work mainly deals with homogeneous networks. WBANs primarily operate in the license-free industrial, scientific, and medical band centered at 2.45 GHz. This is an overcrowded radio band, which is shared with other major wireless standards such as IEEE 802.11, Bluetooth, and cordless phones. Studies regarding coexistence issues with other wireless technologies operating at 2.45 GHz can be found in [3]. In [11], the authors reviewed studies on the coexistence between IEEE 802.11 and IEEE 802.15.4-based networks following the method of question analysis solution. In [12], a coexistence structure to ensure WBANs robustness to Wi-Fi interference was presented. Extensive efforts have been made to enhance the performance of coexisting WBANs. In [13], the authors used stochastic geometry analysis to develop spectrum-efficient multi-channel random wireless networks. Using a graph theory, they obtained the minimum required number of channels needed to accommodate a given intensity of coexisting networks under an outage probability constraint.

### Power Control

Two main approaches, namely, optimization and game theory approaches, have been used to analyze and design sensible power control policies in wireless networks [14]. Advances in wireless sensor networks, such as power supply miniaturization, increased battery duration, reduced energy consumption, and power scavenging, are essential to systems that undertake pervasive monitoring, particularly in regard to implantable sensors [15]. In [16], the authors developed a class of practical online schemes that dynamically adapt transmission power based on receiver feedback. Some other papers have investigated power optimization for MAC protocols [17]- [20]. In [21], a distributed queuing WBANs MAC protocol commitment was proposed to guarantee that all packet transmissions are served with their particular application-dependent quality-of-service (QoS) requirements.

Some papers dealt with energy efficiency by optimizing node coverage in wireless sensor networks [22]- [23]. Reference [22] proposed an effective route algorithm to preserve k-coverage and showed that it can provide energy consumption with greater efficiency. A novel algorithm, namely, complex alliance strategy with multi-objective optimization of coverage, was introduced in [23]. This method improves the quality of network coverage and mitigates rapid node energy consumption. Reference [24] presented a modified stable election protocol, which organizes the advanced nodes and selects a cluster head, to prolong the network lifetime by maintaining balanced energy consumption. These algorithms achieve effective coverage and energy efficiency in the same network; however, problems among networks have not yet been considered.

Many studies have already noted the effect of carrier-sensing range optimization in the IEEE 802.11 standard. In [25], the effect of considering order-dependent capture of the optimal choice of a carrier-sensing range in IEEE 802.11a wireless ad hoc networks with decentralized control was investigated. The work in [26] indicated that virtual carrier-sensing is far from being sufficient to solve interference problems and that a larger physical carrier-sensing range can relieve interference to some extent. Additionally, many HCPP model studies are available.

### Throughput Analysis

The authors of [27] presented an optimal channel assignment in the link-layer protocol that adopts learning automata for channel assignment in wireless mesh networks. This strategy aims to minimize interference in the overall network and thus increases the total throughput. In [28], a fairness-based throughput maximization heuristic algorithm to obtain the throughput optimization among coexisting WBANs was proposed. It estimated and adaptively scheduled wireless resources to meet the throughput requirements of all inter- and intra-BSN links. However, the algorithms of these studies used limited WBANs and did not consider the many network scenarios. A method to adaptively allocate a wireless resource was studied in [29]. It achieved effective inter-BSN information sharing and established inter-BSN links with QoS assurance.

The above-mentioned works mainly focused on the MAC protocol design. However, in our network model, we emphasize power control optimization according to actual network conditions. In contrast to most of the other works listed above, we allow the transmit power in each node to vary in an arbitrary distribution subject to the WBANs coexistence density and under the constraint of carrier-sensing thresholds. The purpose of the strategy proposed in this paper is to improve system throughput using joint carrier-sensing thresholds and power controls based on stochastic geometry.

## System Model

The IEEE 802.15.4-based WBANs may be configured in a star, tree, or mesh topology. Among them, the star topology is suitable for one-hop wireless communication. Most of the previously mentioned WBANs adopt this type of structure because of short-distance communication [3].

**Fig 1:**
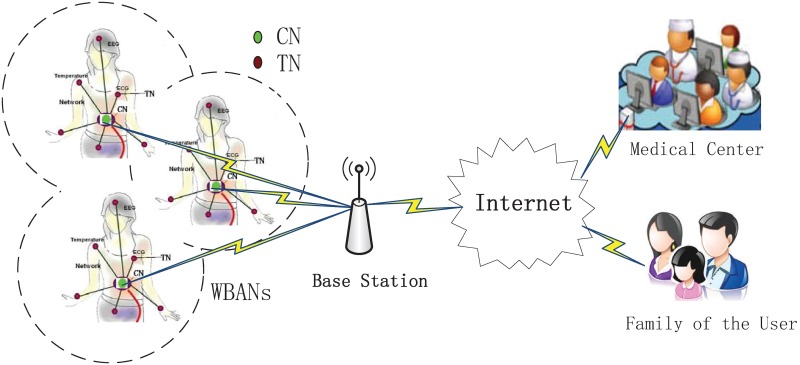
WBANs coexistence model.

The system model consists of multiple stationary WBANs coexisting in an $R^{2}$ Euclidean space, as shown in Fig 1. Each WBAN focuses on the star topology that consists of one coordinator node (CN) and a number of terminal nodes(TNs). According to the IEEE 802.15.4 standard, only one active link exists within any WBAN in the beacon-enabled mode [13]. Hence, we can simplify each WBAN as one TN and a CN to better analyze the inter-WBANs performance. In particular, we let r denote the distance between node TN and CN. Each active TN is considered a PPP with intensity *λ*_*p*_. We denote the target domain of an area by G. By denoting the number of TNs in A by n, the number of TNs in domain *G* is expressed as $\left(\phi \left(G\right)=n\right)=\frac{{\left(\lambda_{p}|G|\right)}^{n}e^{-\lambda_{p}\left|G\right|}}{n!}$, where |*G*| denotes the area of set *G*.

In this paper, all nodes are assumed that they have identical physical layer characteristics and there is no statistical dependence at different nodes. In our model, contention domain is that node *x*_*i*_ is in the contention domain of node *x*_*j*_ if the power received by *x*_*i*_ from *x*_*j*_ is above some detection threshold: $C=\{(x_{i},m_{i},P_{i})\in \tilde{\phi }:P_{i}h||x_{i}-x_{j}|{|}^{-\alpha }\geq \gamma \}$. The time mark *m*_*i*_ corresponds to the backoff timer randomly generated by the carrier-sensing multiple access with collision avoidance (CSMA-CA) protocol for each transmitter. In IEEE 802.15.4 standard, the backoff time is dependent on the *BE* and *aUnitBackoffPeriod*, where *BE* is the backoff exponent which is related to how many backoff periods nodes shall wait before attempting to assess a channel, *aUnitBackoffPeriod*, is the number of symbols forming the basic time period given by 20 × 16 *μs* [7]. The node begins transmitting its data only for it having the lowest mark in their contention domains. The another node sends data packets in the next time slot according to the superframe scheduling.

This PPP model is denoted by $\tilde{\phi }=\left\{\left(x_{i},m_{i},P_{i}\right)\right\}$, where

1. *ϕ* = {*x*_*i*_} denote the locations of the TNs, *ϕ* is always assumed to be Poisson distribution with a positive and finite intensity *λ*_*p*_.
2. *m*_*i*_ are i.i.d. marks, uniformly distributed on [0, 1], according to [0,*Back off timer*/(320 · (2^*BE*^ − 1))], *BE* ∈ [0, 5] [7].
3. *P*_*i*_ denotes the power transmitted of node i.

We assume that the radio signal propagation follows the log-distance path model and ignore shadowing and fast fading with a path loss exponent *α*(*α* > 2). Hence, received power *P*_*r*_ at distance *d* from TN to CN is *P*_*t*_*hd*^−*α*^, where random variable *h* follows an exponential distribution with mean $\frac{1}{\mu }$, which we denote as *h* = exp(*μ*).

## Joint Carrier-sensing Threshold and Power Control

We propose a new joint carrier-sensing threshold and power control mechanism based on PPP model in order to derive both an appropriate carrier-sensing range and a transmission range, which can reduce competition and collision in coexisting WBANs. This approach is suitable for wireless sensor networks or ad hoc networks. This strategy includes four steps. First, we analyze the performance of coexisting WBANs and derive the concept of joint power control according to the nodes distribution in order to reduce the inter-interference. Second, we obtain an expression for the probability density function (PDF) of the transmit power under the carrier-sensing threshold. Thirdly, we compute the success probability according to the SINR and develop an expression for the throughput. Fourthly, we optimize the carrier-sensing threshold and transmit power to obtain the maximum throughput.

### Analysis of the Physical Carrier-sensing Threshold of the CSMA Mechanisms

In this section, we focus on the physical carrier-sensing mechanism. The medium access mechanism for the IEEE 802.15.4 standard uses CSMA-CA and supports the star topologies [7]. Carrier-sensing is a fundamental mechanism in CSMA-CA protocols. In the CSMA-CA mechanism, if the number of wireless nodes increases, data frame collisions occur more often among wireless nodes and lead to a decrease in the total throughput.

In most studies of 802.11 networks, the concept of a carrier-sensing threshold is analyzed [30]. The CSMA-CA mechanism has an inherent limitation in that it suffers from hidden and exposed nodes. It uses static values for the physical carrier- sensing threshold and transmit power, which are not optimum across a range of different scenarios [31]. The transmission range is defined as the maximum distance to a receiver node where the signal can be received at a level above the signal-to-interference-plus-noise ratio (SINR) threshold. The carrier-sensing range delineates the scope where a channel is considered busy depending on the carrier-sensing threshold. In this work, we assume that the carrier-sensing threshold is implemented as an energy detection process within the current channel [30]. Usually, the carrier-sensing range is much larger than the transmission range.

Physical carrier-sensing is a valid mechanism of the CSMA-CA protocols to reduce collisions in wide BSN coexistence, and the carrier-sensing thresholds have a great influence on the network performance. The transmission range is around the core of the sending node, but it is actually related to the receiver. The carrier-sensing range, however, is actually correlated to the transmission. The sending node detects whether the channel is busy depending on the carrier-sensing threshold.

**Fig 2:**
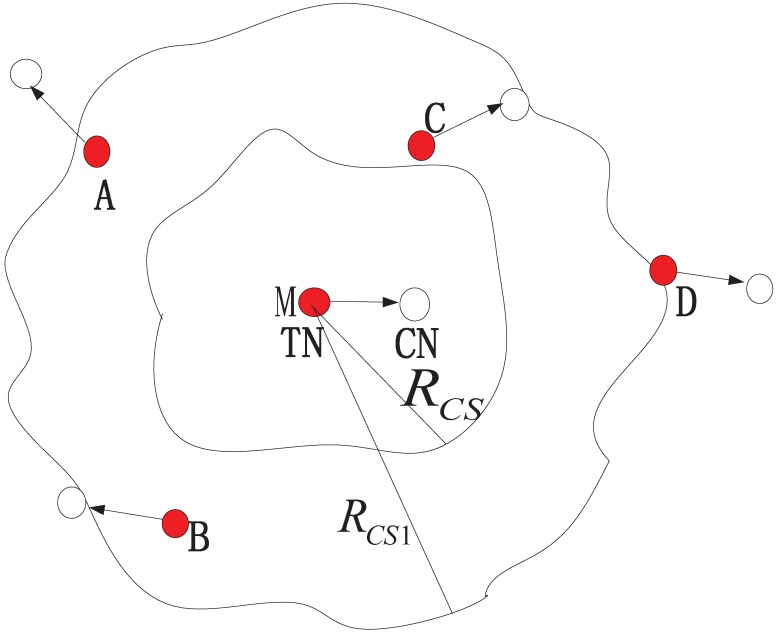
Carrier-sensing range model.

Fig 2 shows the effects of the different carrier-sensing range on the performance of coexisting WBANs. Because of channel fading, no regular-shaped region exists to determine the carrier-sensing range. We consider two carrier-sensing ranges *R*_*CS*_ and *R*_*CS*1_ shown in Fig 2, where *R*_*CS*_ and *R*_*CS*1_. If the carrier-sensing range is *R*_*CS*_, then no other nodes contend for the channel with node M. Decreasing the carrier-sensing range decreases the contention domain of each TN and thereby increases the number of TNs that can share the same channel. Accordingly, the throughput is improved. However, the number of interfering nodes also increases, and the outage probability becomes higher. In this example, nodes A, B, and C in range *R*_*CS*1_ also contend with M. Therefore,the carrier-sensing range has a large effect on the network performance, especially when multi-WBANs coexist.

### Stochastic transmit power control design methodology

Because of the movement of people in coexisting WBANs, the distribution of the nodes cannot be predicted and is thus treated as random. The purpose of the power control mechanism is to the determine the minimum transmit power value that ensures a properly decoded message. Because of the interference of the surrounding nodes, this value is not fixed. In this case, an appropriate carrier-sensing threshold exists. The transmit power value largely depends on networks density and carrier-sensing threshold. The ideal situation is when the carrier-sensing range is relatively small when it crosses into the other domains and yet satisfies the success probability constraints. The simplest case is one point in a circle with a radius of the carrier-sensing range; however, this is not what generally happens. Sometimes, several TNs are present in a circle because of the stochastic distribution of the nodes. Our joint carrier-sensing threshold and power control strategy determines the relationship among the transmit power, carrier-sensing threshold, and total network density. It can not only reduce the total energy consumption of the network but also overcome the main limiting factor of the interference.

First, we derive the PDF for carrier-sensing range. Without loss of generality, because of the stationary of the point process, we may consider node A to be at the origin *o*. The radius of circular M is expressed by carrier-sensing range *R*_*CS*_. The complementary cumulative distribution function (CCDF) of the radius of similar circle M is denoted by *R*_*CS*_, which is given by

$$\left(R_{CS}\geq r\right)=\left(\phi \left(M\right)=1\right)=exp(-\lambda_{p}\left|M|\right)\frac{\lambda_{p}\left|M\right|}{1!}=\lambda_{p}\pi r^{2}\text{exp}\left(-\lambda_{p}\pi r^{2}\right).$$ (1)

Hence, the PDF of *R*_*CS*_ is equal to $f_{R_{CS}}(r)=2\lambda_{p}\pi r(\lambda_{p}\pi r^{2}-1)\text{exp}(-\lambda_{p}\pi r^{2})$. The maximum possible transmission range between a source/destination pair should be less than ${\left(\frac{P_{t}}{\gamma }\right)}^{\frac{1}{\alpha }}$. The distribution of the other TNs those around it is $f_{R_{CS}}(r)$, as expressed in Eq (2), and the *P*_*t*_ = *P*_*th*_(*d*_0_)^*α*^ (0 < *P*_*t*_ < *P*_max_). Therefore, the PDF of *P*_*t*_ is expressed by the following Lemma according to the compound function calculation.

**Lemma 1**. In a coexisting WBANs environment with joint carrier-sensing threshold and minimum power control, for a carrier -sensing threshold *γ* and density distribution *λ*_*p*_, the PDF of the transmit power of a generic active TN is given by

$$\begin{array}{l} f\left(P\right)=\frac{2\lambda_{p}\pi {\left(P\right)}^{\frac{2}{\alpha }-1}}{\alpha {\left(P_{th}\right)}^{\frac{2}{\alpha }}\left(1-\lambda_{p}\pi {\left(\frac{P_{\text{max}}}{P_{th}}\right)}^{\frac{2}{\alpha }}\text{exp}\left(-\lambda_{p}\pi {\left(\frac{P_{\text{max}}}{P_{th}}\right)}^{\frac{2}{\alpha }}\right)\right)}\left[\lambda_{p}\pi {\left(\frac{P}{P_{th}}\right)}^{\frac{2}{\alpha }}-1\right]\text{exp}\left(-\lambda_{p}\pi {\left(\frac{P}{P_{th}}\right)}^{\frac{2}{\alpha }}\right)\\ \;(0<P<P_{\text{max}}). \end{array}$$ (2)

The expected value of the transmit power can be expressed as

$$E\left[P_{t}\right]=\frac{2\lambda_{p}\gamma }{\alpha (1-\lambda_{p}\pi {\left(\frac{P_{\text{max}}}{\gamma }\right)}^{\frac{2}{\alpha }}\text{exp}\left(-\lambda_{p}\pi {\left(\frac{P_{\text{max}}}{\gamma }\right)}^{\frac{2}{\alpha }}\right))}\left[\frac{\alpha }{2}\int_{0}^{P_{\text{max}}}x^{\frac{\alpha }{2}}\text{ exp}\left(-\lambda_{p}\pi x\right)dx-{P_{\text{max}}}^{\frac{\alpha }{2}+1}\text{ exp}\left(-\lambda_{p}\pi P_{\text{max}}\right)\right].$$ (3)

Proof: See **Appendix A**.

### Modeling the Success Probability and Throughput

To correctly receive a packet, the signal at the receiver must be sufficiently sensitive strong enough. Hence, the SINR must be greater than the minimum value specified for the receiver equipment. A packet transmission can be considered successful if the receiving SINR at point y from a transmitter located at point x is greater than or equal to the SINR threshold *β*. We consider *CN*_0_ located at the origin, $CN_{0}\in \tilde{\phi }$, and its receiver node (TN) is located at (*r*_0_, *θ*). We can express the SINR of *CN*_0_ as [32]

$$SINR=\frac{\gamma h}{W+\sum_{x\in \phi_{I}}P_{i}h_{i}\left|\right|x_{i}|{|}^{-\alpha }}.$$ (4)

The number of TNs in set A with radius of carrier-sensing range is a Poisson random variable with mean *λ*_*p*_|*M*| (*λ*_*p*_*πR*_*CS*_^2^). The probability that node A has the lowest mark is $\frac{1}{\lambda_{p}\pi {R_{CS}}^{2}}$. We obtain the probability of A being retained in M as

$$P\left(A\right)=Prob\left\{A\;\;\;\;is\;\;\;\;retained\;\;\;\;in\;\;\;\;M\right\}=\frac{1}{\lambda_{p}\pi {R_{CS}}^{2}}.$$ (5)

**Lemma 2**. With aggregate interference, the success probability of *TN* at a generic time instant can be expressed by the following Lemma:

$$p_{s\_c}=\left(SINR\geq \beta \right)=\text{exp}\left(-\lambda_{p}{\left(\frac{E[P_{t}]}{P(A)\gamma }\right)}^{\frac{2}{\alpha }}\int_{{\left(\frac{s\gamma }{u}\right)}^{\frac{-1}{\alpha }}}^{\infty }\frac{y}{1+y^{\alpha }}dy\right).$$ (6)

The throughput of the coexisting WBANs is defined as the maximum spatial intensity of successful transmissions in a certain domain under the condition that the outage probability does not exceed some specified threshold. We let *S*_*c*_ be the normalized throughput of the coexisting networks under power control, defined as the average time of successfully transmitted payload bits. In this section, we can approximate the throughput as

$$S_{c}=\frac{\lambda_{p}p_{s\_c}L}{T},$$ (7)

where L is the length of the transmit packet and T (*T* = *T*_*t*_ + *T*_*sense*_ + *T*_*back*_ + *T*_*idle*_) is the average time to successfully send a packet. *T*_*t*_, *T*_*sense*_, *T*_*back*_ and *T*_*idle*_ represent the total time for a radio to transmit, time taken to sense the channel, time needed to backoff, and idle time, respectively.

Considering all these factors, we will optimize the carrier-sensing threshold to increase the throughput as much as possible.

$$\begin{array}{l} \text{maxmize }\frac{\lambda_{p}p_{s\_c}L}{T}\\ subject\;to\;p_{s\_c}>\sigma \\ \gamma_{\text{min}}<\gamma <\gamma_{\text{max}}, \end{array}$$ (8)

where *p*_*s*_*c*_ > *σ* is the success probability and *σ* is minimum constraint value of success probability.

## Throughput of Coexisting Networks with Guard Zones

In CSMA, all the nodes in the network contend for the channel. Transmitter nodes can attempt to simultaneously use the same channel in coexisting WBANs, which results in mutual interference. To effectively reduce the intensity of the interference signal, a protection area in the coexisting WBANs proposed around each node, where no other transmitter node inside is allowed to transmit data. The guard zone can be modeled as a type II HCPP with a hard-core size of influence that is optimized using stochastic geometry. Because the HCPP model is a particular thinning of a homogeneous PPP model according to *m*_*i*_ of nodes *x*_*i*_, the distance between two selected nodes is always greater than the hard-core size [33].

Each time a node desires to transmit data frames or MAC commands, it first waits for a backoff period. Following a random backoff, if the channel is found to be idle, the device transmits its data. If the channel is found to be busy following a random backoff, the node waits for another random period before trying to access the channel again [14]. Throughout this paper, we define the backoff time as mark *m*. The node with the smallest marks will transmit its packet first. The model guard zone retains only nodes with the lowest mark in their contention domains. Therefore, the density of the model with guard zones based on the PPP model is given by *λ*_*h*_ Eq (10). According to mark t, the probability of being chosen is

$$P_{h}=E[{\left(1-m\right)}^{n}]=\frac{\left(1-e^{-\lambda_{p}\pi z^{2}}\right)}{\lambda_{p}\pi z^{2}}.$$ (9)

Therefore, the final density of the HCPP model with a guard zone is

$$\lambda_{h}=\lambda_{p}\frac{\left(1-e^{-\lambda_{p}\pi z^{2}}\right)}{\lambda_{p}\pi z^{2}}=\frac{\left(1-e^{-\lambda_{p}\pi z^{2}}\right)}{\pi z^{2}},$$ (10)

where z denotes the size of the guard zone, which is the radius of the circle around each node. The left node has a minimum mark in the same circle.

$$p_{s\_h}=\text{exp}\left(-\frac{\beta ur^{\alpha }W}{P_{t}}\right)\text{exp}\left(-\lambda_{h}d\left(\alpha \right){\left(\frac{\beta }{u}\right)}^{2/\alpha }r^{2}\right).$$ (11)

Proof: See **Appendix B**.

Additionally, the success probabilities of the PPP and HCPP models are proven in **Appendix C**.

According to success probability *p*_*s*_*h*_, the throughput can be expressed as

$$S_{h}=\frac{\lambda_{h}p_{s\_h}L}{T}=\frac{\left(1-e^{-\lambda_{p}\pi z^{2}}\right)\text{exp}\left(-\lambda_{h}C\left(\alpha \right){\left(\frac{\gamma }{u}\right)}^{2/\alpha }r^{2}\right)L}{\pi z^{2}T},$$ (12)

and we can obtain the following non linear optimization problem:

$$\begin{array}{ll} \text{maxmize} & \frac{\lambda_{h}p_{s\_h}L}{T}\\ subject\;to & p_{s\_h}>\sigma . \end{array}$$ (13)

Following a method similar to the Lagrange duality approach, we define

$$g\left(z\right)=S\left(z\right)+\zeta (p_{s\_h}\left(\beta ,z\right)-\sigma ).$$ (14)

According to *d*(*g*(*z*))/*d*(*z*), the optimized value is obtained as z = 0.36.

## Performance Analysis

### Comparing the Joint Carrier-sensing Threshold and Power Control with the HCPP Model

By analyzing the conditions for competition prevention, we argue that for the CSMA protocols to adapt to changes in the distribution of coexisting networks, the carrier-sensing threshold should be as small as possible, and the transmit power should accordingly change. We have discussed the throughputs of the joint carrier-sensing threshold and power control scheme, the ordinary PPP model, and the HCPP model. The ordinary PPP model is the poorest performer among all strategies. Although both the joint power control and HCPP model mechanisms adjust the competition area to ensure the throughput, their approaches are very different.

The joint power control approach finds the most suitable carrier-sensing range and transmits power among many star WBANs. On the other hand, the HCPP model mainly applies to multiple nodes in one network that usesa CSMA mechanism where only one node can successfully transmit at a time in each star network. The HCPP models form a generic class of point processes whose points are never closer to each other than some given distances [34]. A node is retained if it has the minimal backofftime among all nodes in its contention domain; otherwise, it is thinned out. The main drawback of the HCPP model is that it does not support transmitters that are very close. Thus,the thinning process can lead to an inaccurate performance analysis of the system [35]. However, the analysis of the joint power control mechanism does not modify the network distribution. Its purpose is to optimize the transmit power and carrier-sensing range under channel gains to maximize the throughput. The joint power control can ensure more practical results.

### Energy Efficiency

An accurate and comprehensive energy efficiency model forms the basis of power consumption analysis in energy-efficient system design. Energy efficiency is defined as the ratio of the average number of bits transmitted to the power consumed and has a unit of bps/Hz/W [13].

$$\eta_{EE}=\frac{S}{(\frac{\xi }{\varsigma }P_{t}+P_{c})T}$$ (15)

In Eq (15), *ξ* is the peak-to-average power ratio (PARR), which is the square of the peak amplitude (given the peak power) divided by the root mean square. A 2450-MHz DSSS PHY employing O-QPSK modulation with half-sine pulse shaping results in a PARR of 1.4. *ζ* is the drain efficiency of the PA. It is less than 10%. Therefore, the total circuit power must be considered. Pt is the transmit power, and Pc is the power consumed in various transmit and receive operations in the electronic circuit, excluding the PA power. Circuit power Pc affects the energy consumption and, hence, the energy efficiency. It is also a key design parameter.

The energy efficiency of the joint power control strategy and HCPP system are much higher than that of the ordinary PPP model because they minimize competition and interference. If the value of the guard zone is appropriate, the energy efficiency of the HCPP model is higher than that of the joint power control strategy due to the lack of nodes.

## Performance Evaluation

The aim of this section is to verify the throughput analysis through a MATLAB simulation. In the simulation, a CN and a TN form a WBAN. The maximum transmission distance between the coordinator and the sensor node in the BAN is set to 5m. The radio settings are configured according to the IEEE 802.15.4 standard. In this study, we consider an on-body path-loss model with exponent and with the parameters listed in Table 1.

**Table 1:** System Parameters.

Parameters	Value
Maximum value of transmit power *P*_*max*_ (mW)	1
On-body path-loss exponent *α*	3
threshold *β* (dB)	-20
Carrier sensing threshold *γ*_*min*_ (dBm)	-80
Carrier sensing threshold *γ*_*max*_ (dBm)	-30
Channel bandwidth *B* (Mhz)	2
Frequency(Ghz)	2.4
Channel rate *R* (kbps)	200
Symbol time(us)	16
Backoff exponent *BE*	3
aUnitBackoffPeriod	20
Circuit power *P*_*c*_ (mW)	30

**Fig 3:**
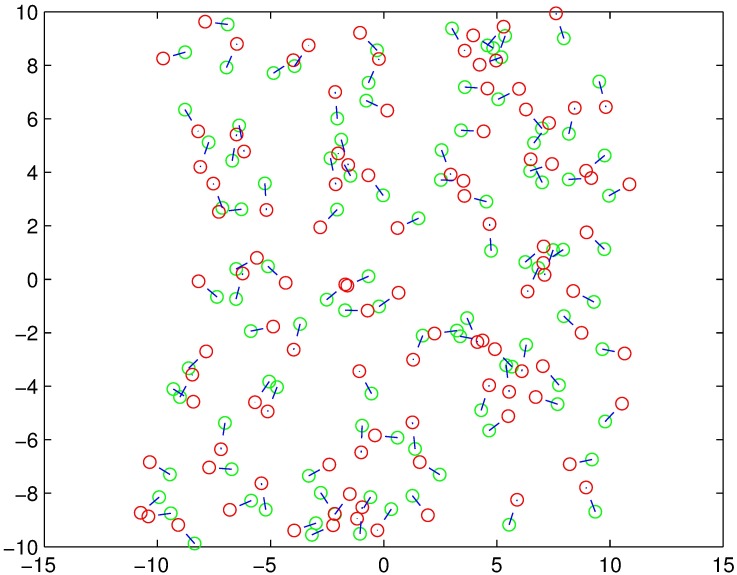
PPP model (Red dots represent the CNs while the green dots represent the TNs).

**Fig 4:**
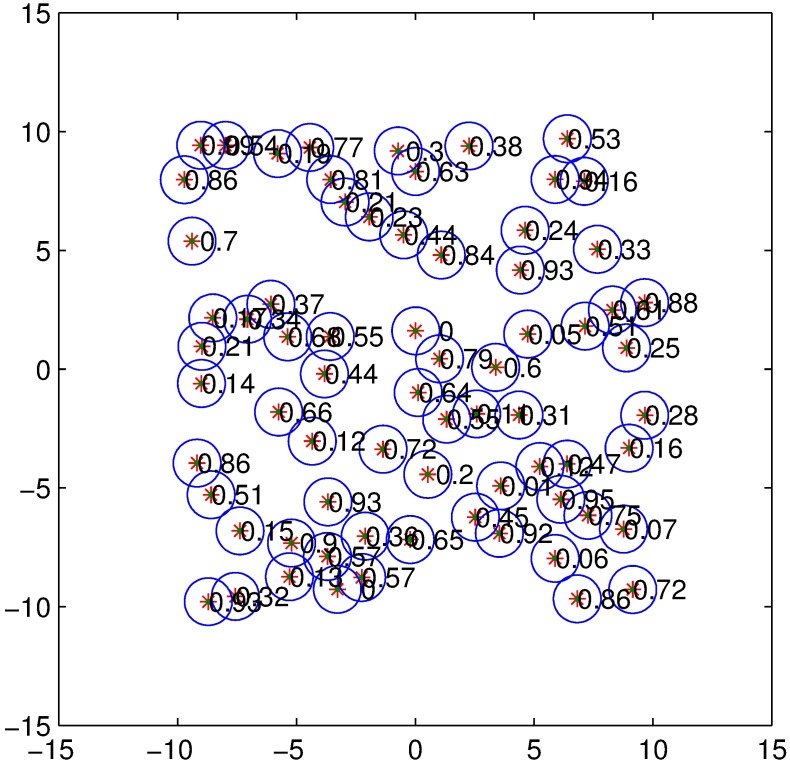
HCPP model.

We assume that all nodes, including both the CNs and TNs, have the same transmit parameters except for the transmit power and carrier-sensing threshold. The effective transmission range changes with the carrier-sensing threshold. The contention domain is the carrier-sensing range of each transmitter. Because transmission nodes are distributed in a 20m ×20m area according to a certain density such as Fig 3, to simplify the analysis, we exploit the fact that according to the CSMA standard, only one node sends data at a time in each WBAN. Fig 4 shows an HCPP model with the hard-core guard zone set to 1m. The HCPP model describes patterns that include only the minimal mark retained in the threshold of the guard zone. The center of the circle represents a TN, whereas the decimal denotes the value of the mark. The radius of each circle represents the size of the hard-core zone.

**Fig 5:**
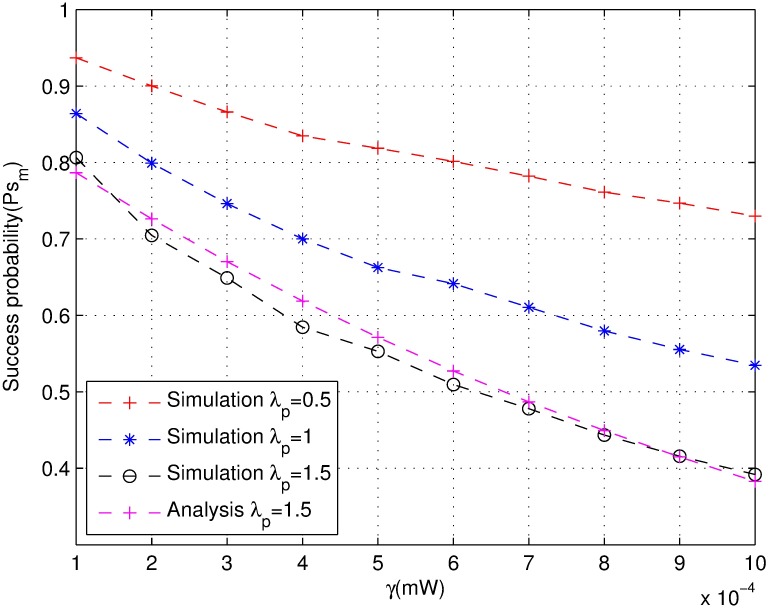
The success probability under different carrier-sensing threshold *γ*.

**Fig 6:**
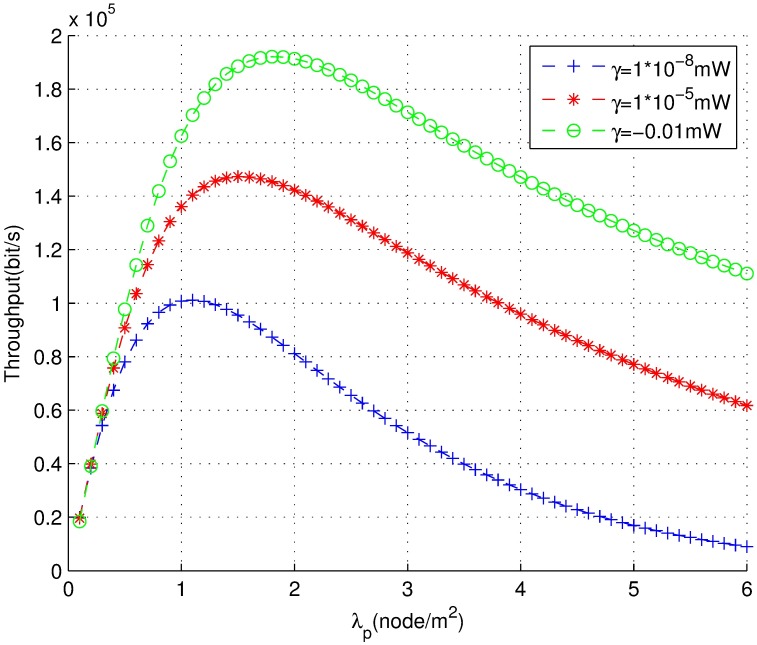
The throughput under different carrier-sensing threshold *γ*.

The relationship between carrier-sensing threshold value *γ* and transmission success probability *p*_*s*_*c*_ is shown in Fig 5. The simulation is repeated 10000 times. The success probability of joint power control declines with the increasing of the carrier-sensing threshold [30]. This result suggests that the interference level and the outage probability increase with the carrier-sensing range decreasing. However, it increases the throughput and the spatial frequency reuse such as that shown in Fig 6. The throughput at *γ* = 1 × 10^−8^*mW* is almost two times as much as *γ* = 0.01*mW* at the peak value. On the other hand, the throughput monotonically increase more slowly with density *λ*_*p*_ than it does with a different value under a different *γ* and then slowly declines. Therefore, the carrier-sensing range optimization is closely related to the density of the network for optimizing the throughput.

In addition, each of the above listed three figures shows that the larger the carrier-sensing range is (which also means the carrier-sensing threshold is smaller), the bigger will be the contention domain. The success probability and the throughput are also larger. In other words, the smaller the carrier-sensing threshold is, the higher is the throughput, and vice versa.

**Fig 7:**
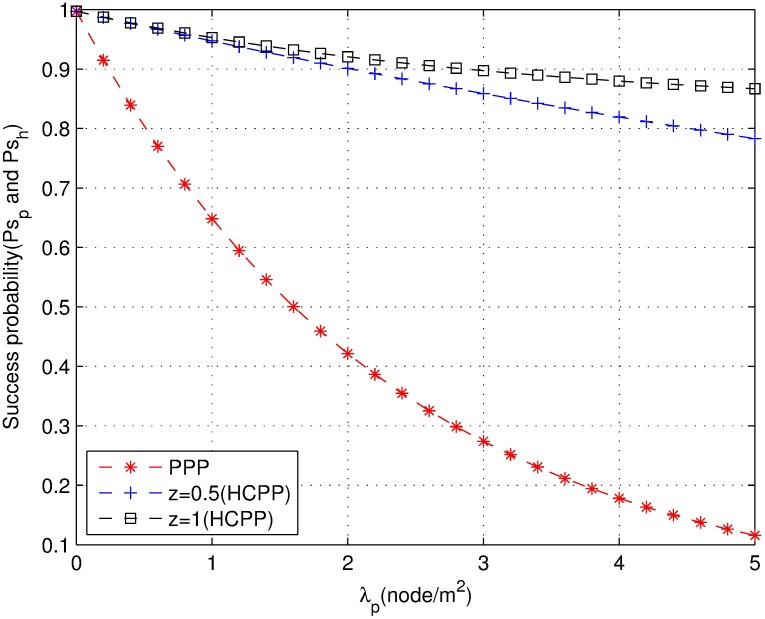
The success probability with the guard zone.

Fig 7 shows the comparison of the success probability of the PPP and HCPP models under the same density *λ*_*p*_. The success probability of the HCPP model with a guard zone is significantly higher than that of the PPP model. Indeed, the success probability of the HCPP model is always higher than 0.7. Moreover, the bigger the z is, the higher is the success probability. The PPP model quickly degrades as the density increases, which suggests that the HCPP with the guard zone model naturally decreases the interference. Therefore, an optimal guard zone can enhance the performance of coexisting networks.

**Fig 8:**
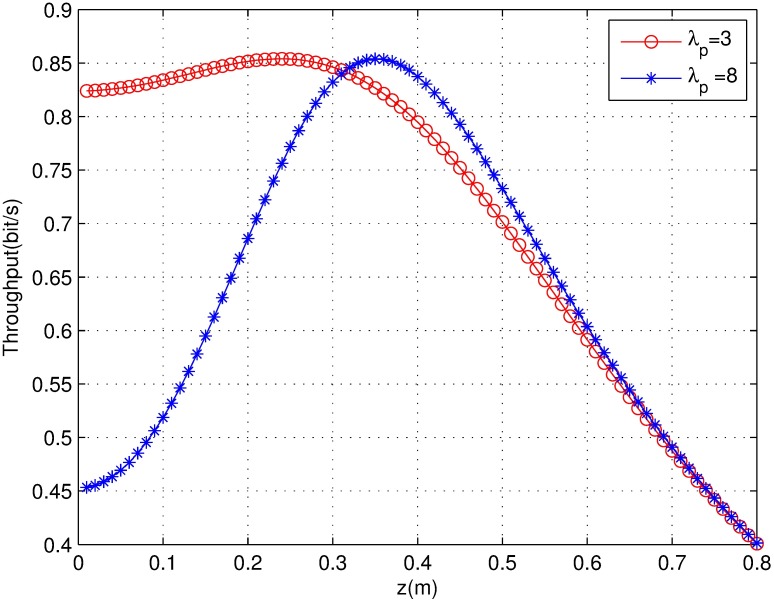
The throughput of HCPP model with guard zone.

The throughput simulation result of the HCPP model is shown in Fig 8. This figure shows that the throughput will decrease when the network coexistence is greater than a certain density because when the density is large, the number of conflicts increases and the interference becomes high. In particular, when the network density is greater than a certain value, the throughput irregularly changes. The throughput is low when the size of the guard zone is relatively small or very large. From this figure, we observe an optimal throughput at 0.3 < z < 0.4, which is consistent with the analysis result z = 0.36.

**Fig 9:**
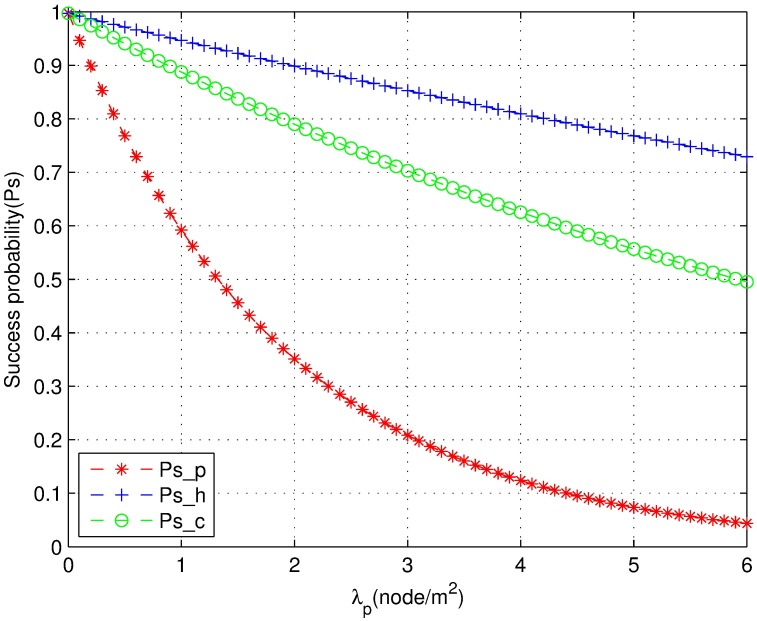
The success probability comparison of three mechanisms.

**Fig 10:**
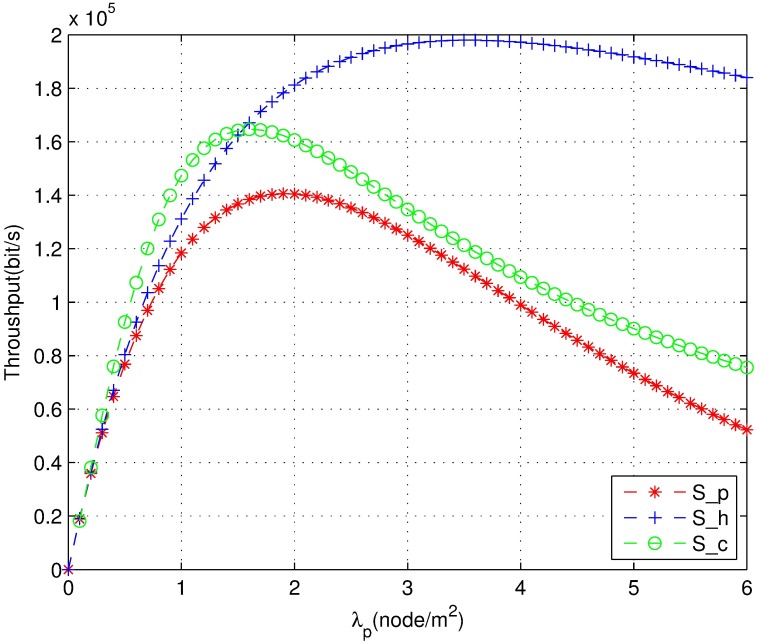
The throughput comparison of three mechanisms.

Fig 9 shows the comparison of the success probability of the joint power control mechanism, PPP model, and HCPP model under different node densities *λ*_*p*_. The performance of the proposed joint power control mechanism and HCPP model is superior to that of the PPP model. The throughput of the three strategies is compared in Fig 10. The throughput of the joint power control mechanism is superior to that of the HCPP model when the density is lower than two. The reason is that the number of thinning nodes is lower in the HCPP model at lower densities. Hence, the curve of the HCPP model is much flatter than the joint power control curve. However, the joint power control mechanism is more realistic. The better performance of the HCPP model is due to the lower density through thinning of a number of nodes. The energy efficiency is similar to that shown in Fig 10. From this analysis and simulations, we can obtain the appropriate density to provide a reference to carry out a standard or system design. We obtain the closed expression for success probability and the throughput through transmission power control and optimization. Therefore, the time complexity of this algorithm is O(1) according to Eqs (2) and (6) [36].

## Conclusion

Carrier-sensing threshold is a very critical parameter that should be carefully chosen to maintain the balance between the throughput and success probability. Owing to the limited battery capacity in sensor nodes, effective power control is especially crucial in the design and performance of WBANs. In this paper, we have proposed a new power control mechanism that depends on the least carrier-sensing threshold. Throughput is guarantied through this strategy and HCPP modeling. A transmitter can adapt its power level based on the propagation loss and interference of the surrounding nodes to its intended receiver. The results show that throughput is largely dependent on the network distribution density (*λ*_*p*_), transmit power of the nodes (*P*_*t*_) and carrier-sensing threshold (*γ*). We have obtained an approximate expression for the success probability and throughput. Although lowering the power level is beneficial, particularly for battery-operated nodes, the primary objective is to maximize the throughput of coexisting WBANs using a joint power control strategy. A turning point of the throughput exists under a certain density.

In addition, we modeled the system by adopting HCPP and analyzed the effect of the guard zone size on the network throughput. We show that setting a reasonable size of the guard zone can greatly reduce interference in the network, increase the success probability, and optimize the throughput. Moreover, the energy consumption and spectral efficiency are improved. Finally, we compare the similarities and differences between these two strategies.

WBANs often run in higher density networks than the traditional wireless cellular networks and require stricter power management to reduce the energy consumption and interference [29]. Our framework is designed for dense WBANs scenarios and helps promote WBANs application. Future investigations are needed to test and verify the proposed strategies in an actual system.

## Appendix

### Appendix A

We can get the minimum transmission power as much as possible to reduce interference to the surrounding nodes. The transmit power of TNs is expressed as *P_t_* = *γr*_0_^*α*^, where the *r*_0_ is the distance of source/destination pair. The carrier-sensing range distribution of TNs is $f_{R_{CS}}(r)=2\lambda_{p}\pi r(\lambda_{p}\pi r^{2}-1)\;\text{exp}(-\lambda_{p}\pi r^{2})$. So, the PDF of transmit power is given by

$$\begin{array}{l} g(P)\underline{\underline{\left(a\right)}}2\lambda_{p}\pi {\left(\frac{P}{P_{th}}\right)}^{\frac{1}{\alpha }}[\lambda_{p}\pi {\left(\frac{P}{P_{th}}\right)}^{\frac{2}{\alpha }}-1]\text{exp}(-\lambda_{p}\pi {\left(\frac{P}{P_{th}}\right)}^{\frac{2}{\alpha }})\frac{d({\left(\frac{P}{P_{th}}\right)}^{\frac{1}{\alpha }})}{d(r)}\\ \;=2\lambda_{p}\pi {\left(\frac{P}{P_{th}}\right)}^{\frac{1}{\alpha }}[\lambda_{p}\pi {\left(\frac{P}{P_{th}}\right)}^{\frac{2}{\alpha }}-1]\text{exp}(-\lambda_{p}\pi {\left(\frac{P}{P_{th}}\right)}^{\frac{2}{\alpha }})\frac{1}{\alpha }{\left(\frac{P}{P_{th}}\right)}^{\frac{1}{\alpha }-1}\\ \;=\frac{2\lambda_{p}\pi {\left(P\right)}^{\frac{2}{\alpha }-1}}{\alpha {\left(P_{th}\right)}^{\frac{2}{\alpha }}}[\lambda_{p}\pi {\left(\frac{P}{P_{th}}\right)}^{\frac{2}{\alpha }}-1]\text{exp}(-\lambda_{p}\pi {\left(\frac{P}{P_{th}}\right)}^{\frac{2}{\alpha }}) \end{array}$$ (16)

$\underline{\underline{\left(a\right)}}$ is obtained by the calculation of probability density function of composite function.

$$\begin{array}{c} \int_{0}^{P_{\text{max}}}g\left(x\right)dx\\ =\int_{0}^{P_{\text{max}}}\frac{2\lambda_{p}\pi {\left(x\right)}^{\frac{2}{\alpha }-1}}{\alpha {\left(P_{th}\right)}^{\frac{2}{\alpha }}}\left[\lambda_{p}\pi {\left(\frac{x}{P_{th}}\right)}^{\frac{2}{\alpha }}-1\right]\text{exp}\left(-\lambda_{p}\pi {\left(\frac{x}{P_{th}}\right)}^{\frac{2}{\alpha }}\right)dx\\ =1-\lambda_{p}\pi {\left(\frac{P_{\text{max}}}{P_{th}}\right)}^{\frac{2}{\alpha }}\text{exp}\left(-\lambda_{p}\pi {\left(\frac{P_{\text{max}}}{P_{th}}\right)}^{\frac{2}{\alpha }}\right) \end{array}$$ (17)

$$\begin{array}{c} f\left(P\right)\;\underline{\underline{\left(b\right)}}\;\frac{g\left(P\right)}{\int_{0}^{P_{\text{max}}}g\left(x\right)dx}\\ \begin{array}{c} =\frac{2\lambda_{p}\pi {\left(P\right)}^{\frac{2}{\alpha }-1}}{\alpha {\left(P_{th}\right)}^{\frac{2}{\alpha }}\left(1-\lambda_{p}\pi {\left(\frac{P_{\text{max}}}{P_{th}}\right)}^{\frac{2}{\alpha }}\text{exp}\left(-\lambda_{p}\pi {\left(\frac{P_{\text{max}}}{P_{th}}\right)}^{\frac{2}{\alpha }}\right)\right)}\left[\lambda_{p}\pi {\left(\frac{P}{P_{th}}\right)}^{\frac{2}{\alpha }}-1\right]\text{exp}\left(-\lambda_{p}\pi {\left(\frac{P}{P_{th}}\right)}^{\frac{2}{\alpha }}\right)\;0<P<P_{\text{max}} \end{array} \end{array}$$ (18)

$\underline{\underline{\left(b\right)}}$ is normalized due to the range value of power.

The expected value of transmit power is

$$\begin{array}{l} E[P]=\int_{0}^{P_{\text{max}}}Pf(P)dP\\ \begin{array}{l} =\int_{0}^{P_{\text{max}}}P\frac{2\lambda_{p}\pi {\left(P\right)}^{\frac{2}{\alpha }-1}}{\alpha {\left(\gamma \right)}^{\frac{2}{\alpha }}(1-\lambda_{p}\pi {\left(\frac{P_{\text{max}}}{\gamma }\right)}^{\frac{2}{\alpha }}\text{exp}(-\lambda_{p}\pi {\left(\frac{P_{\text{max}}}{\gamma }\right)}^{\frac{2}{\alpha }}))}[\lambda_{p}\pi {\left(\frac{P}{\gamma }\right)}^{\frac{2}{\alpha }}-1]\text{exp}(-\lambda_{p}\pi {\left(\frac{P}{\gamma }\right)}^{\frac{2}{\alpha }})\;dP \end{array}\\ \begin{array}{l} =\frac{\lambda_{p}\alpha \pi {\left(\gamma \right)}^{\frac{2}{\alpha }+1}}{\alpha {\left(\gamma \right)}^{\frac{2}{\alpha }}(1-\lambda_{p}\pi {\left(\frac{P_{\text{max}}}{\gamma }\right)}^{\frac{2}{\alpha }}\text{exp}(-\lambda_{p}\pi {\left(\frac{P_{\text{max}}}{\gamma }\right)}^{\frac{2}{\alpha }}))}[\frac{\alpha }{2}\int_{0}^{{\left(\frac{P_{\text{max}}}{\gamma }\right)}^{\frac{2}{\alpha }}}x^{\frac{\alpha }{2}}e^{-\lambda_{p}\pi x}dx-{\left(\frac{P_{\text{max}}}{\gamma }\right)}^{1+\frac{1}{\alpha }}\times exp(-\lambda_{p}\pi {\left(\frac{P_{\text{max}}}{\gamma }\right)}^{\frac{2}{\alpha }})] \end{array} \end{array}$$ (19)

### Appendix B

The success probability of joint carrier-sensing threshold and power control can be estimated as

$$\begin{array}{l} p_{s\_c}=\left(SINR\geq \beta \right)\\ =\left(\frac{P\left(A\right)\gamma h}{W+\sum_{x\in \phi_{I}}P_{i}h_{i}||x_{i}|{|}^{-\alpha }}\geq \beta \right)\\ =\{h\geq \left(\frac{\beta \mu W}{P\left(A\right)\gamma }+\frac{\beta \mu }{P\left(A\right)\gamma }\sum_{x\in \phi_{L}}P_{i}h_{i}||x_{i}|{|}^{-\alpha })\right\}\\ =\text{exp}\left(-\frac{\beta \mu W}{P\left(A\right)\gamma }\right)L_{c}\left(s\right)){|}_{s=\frac{\beta \mu }{P\left(A\right)\gamma }} \end{array}$$ (20)

The Laplace trans PDF of the channel gains is expressed by:

$$\begin{array}{l} L_{c}\left(s\right)={\left(1+\frac{s}{u}\right)}^{-1}\\ =E\left(\text{exp}\left(-s\sum_{x\in \phi_{L}}P_{t}h_{i}||x_{i}|{|}^{-\alpha }\right)\right)\\ =E\prod_{x\in \phi_{L}}\text{exp}(-sP_{t}h_{i}|\left|x_{i}\right|{|}^{-\alpha })\\ =E\prod_{x\in \phi_{L}}\frac{1}{1+\frac{s}{u}P_{t}h_{i}||x_{i}|{|}^{-\alpha }} \end{array}$$ (21)

using the (Probability generating functional) PGFL of a PPP [29].

$$\begin{array}{l} E\prod_{x\in \phi_{L}}f\left(x\right)=\text{exp}\left(-\lambda_{p}\underset{R^{2}}{\int }(1-f\left(x\right)dx\right)\\ L_{c}\left(s\right)=\text{exp}\left(-\lambda_{p}\int_{R^{2}}(1-\frac{1}{1+\frac{s}{u}E\left[P_{t}\right]||x_{i}|{|}^{-\alpha }}dx\right))\\ =\text{exp}\left(-\lambda_{p}\int_{{\left(\frac{P_{t}}{\gamma }\right)}^{\frac{1}{\alpha }}}^{\infty }\frac{1}{1+{\left(\frac{s}{u}E\left[P_{t}\right]\right)}^{-1}r_{i}{}^{\alpha }}rdr\right) \end{array}$$ (22)

$$L{}_{c}\left(s\right)=\text{exp}\left(-\lambda_{p}{\left(\frac{sE[P_{t}]}{u}P\left(A\right)\right)}^{\frac{2}{\alpha }}\int_{{\left(\frac{s\gamma }{u}\right)}^{\frac{-1}{\alpha }}}^{\infty }\frac{y}{1+y^{\alpha }}dy\right),$$ (23)

where,

$$y={\left(\frac{s}{u}E\left[P_{t}\right]P\left(A\right)\right)}^{-\frac{1}{\alpha }}r,$$ (24)

$$d\left(y\right)=\frac{1}{\sqrt{3}}\arctan\frac{2y-1}{\sqrt{3}}-\frac{1}{6}\ln\frac{{\left(1+y\right)}^{2}}{1-y+y^{2}},$$ (25)

then,

$$p_{s\_c}=\left(SINR\geq \beta \right)=\text{exp}{\left(-\lambda_{p}{\left(\frac{E[P_{t}]}{P(A)\gamma }\right)}^{\frac{2}{\alpha }}d\left(y\right)\right|}_{{\left(\beta \right)}^{\frac{-1}{\alpha }}}^{\infty }.$$ (26)

### Appendix C

The success probability of PPP moel can be estimated as

$$\begin{array}{l} p_{s\_p}=P\left(\frac{P_{t}hr^{-\alpha }}{W+I}\geq \beta \right)\\ =(h>\left(\frac{\beta r^{\alpha }\left(W+I\right)}{P_{t}}\right))\\ =\left(\text{exp}\left(-\frac{\beta ur^{\alpha }\left(W+I\right)}{P_{t}}\right)\right)\\ =\text{exp}\left(-\frac{\beta ur^{\alpha }W}{P_{t}}\right)\text{exp}\left(-\frac{\beta ur^{\alpha }I}{P_{t}}\right)\\ =\text{exp}\left(-\frac{\beta ur^{\alpha }W}{P_{t}}\right)L\left(s\right){|}_{s=\frac{\beta ur^{\alpha }}{P_{t}}}\\ =\text{exp}\left(-\frac{\beta ur^{\alpha }W}{P_{t}}\right)\text{exp}\left(-\lambda_{p}\left(\frac{2\pi^{2}}{\alpha \text{sin}\left(\frac{2\pi }{\alpha }\right)}\right){\left(\frac{\beta }{u}\right)}^{2/\alpha }r^{2}\right) \end{array}$$ (27)

Then,

$$p_{s\_p}=\text{exp}\left(-\frac{\beta ur^{\alpha }W}{P_{t}}\right)\text{exp}\left(-\lambda_{p}\left(\frac{2\pi^{2}}{\alpha \sin(\frac{2\pi }{\alpha })}\right){\left(\frac{\beta }{u}\right)}^{2/\alpha }r^{2}\right).$$ (28)

In a similar manner, the success probability of HCPP model is written by

$$p_{s\_h}=\text{exp}\left(-\frac{\beta ur^{\alpha }W}{P_{t}}\right)\text{exp}\left(-\lambda_{h}\int_{{\left(\beta \right)}^{\frac{-1}{\alpha }}Z}^{\infty }\frac{y}{1+y^{\alpha }}dy{\left(\frac{\beta }{u}\right)}^{2/\alpha }r^{2}\right).$$ (29)
